# The effect of saliva on the fate of nanoparticles

**DOI:** 10.1007/s00784-017-2172-5

**Published:** 2017-07-09

**Authors:** Birgit J. Teubl, Biljana Stojkovic, Dominic Docter, Elisabeth Pritz, Gerd Leitinger, Igor Poberaj, Ruth Prassl, Roland H. Stauber, Eleonore Fröhlich, Johannes G. Khinast, Eva Roblegg

**Affiliations:** 1 Institute of Pharmaceutical Sciences, Department of Pharmaceutical Technology and Biopharmacy, University of Graz, Universitätsplatz 1, 8010 Graz, Austria; 2 BioTechMed, 8010 Graz, Austria; 3 Faculty of Mathematics and Physics, University of Ljubljana, 1000 Ljubljana, Slovenia; 4 Department of Nanobiomedicine, Mainz University Medical Center, 55131 Mainz, Germany; 5 Institute of Cell Biology, Histology and Embryology, Research Unit Electron Microscopic Techniques, Medical University of Graz, 8010 Graz, Austria; 6 Institute of Biophysics, Medical University of Graz, 8010 Graz, Austria; 7 Center for Medical Research, Medical University of Graz, 8010 Graz, Austria; 8 Institute for Process and Particle Engineering, Graz University of Technology, 8010 Graz, Austria; 9 Research Center Pharmaceutical Engineering, 8010 Graz, Austria

**Keywords:** Nanoparticles, Saliva, Mucoglycoproteins, Biological barrier, Mobility

## Abstract

**Objectives:**

The design of nanocarriers for local drug administration to the lining mucosa requires a sound knowledge of how nanoparticles (NPs) interact with saliva. This contact determines whether NPs agglomerate and become immobile due to size- and interaction-filtering effects or adsorb on the cell surface and are internalized by epithelial cells. The aim of this study was to examine the behavior of NPs in saliva considering physicochemical NP properties.

**Materials and methods:**

The salivary pore–size distribution was determined, and the viscosity of the fluid inside of the pores was studied with optical tweezers. Distinct functionalized NPs (20 and 200 nm) were dispersed in saliva and salivary buffers and characterized, and surface-bound MUC5B and MUC7 were analyzed by 1D electrophoresis and immunoblotting. NP mobility was recorded, and cellular uptake studies were performed with TR146 cells.

**Results:**

The mode diameter of the salivary mesh pores is 0.7 μm with a peak width of 1.9 μm, and pores are filled with a low-viscosity fluid. The physicochemical properties of the NPs affected the colloidal stability and mobility: compared with non-functionalized particles, which did not agglomerate and showed a cellular uptake rate of 2.8%, functionalized particles were immobilized, which was correlated with agglomeration and increased binding to mucins.

**Conclusion:**

The present study showed that the salivary microstructure facilitates NP adsorption. However, NP size and surface functionalization determine the colloidal stability and cellular interactions.

**Clinical relevance:**

The sound knowledge of NP interactions with saliva enables the improvement of current treatment strategies for inflammatory oral diseases.

**Electronic supplementary material:**

The online version of this article (doi:10.1007/s00784-017-2172-5) contains supplementary material, which is available to authorized users.

## Introduction

The mouth is a well-organized system, often referred as the mirror of the body that reflects and supports human health. It has a variety of functions, which closely work together, to prevent absorption of foreign substances, maintain the oral ecosystem, and support the digestion process [1]. Moreover, it facilitates chewing, biting, speaking, smiling, and, consequently, psychosocial well-being. However, general conditions may change this homeostasis and increase the risk of oral diseases and disorders. For example, infections [2], oral cancer [3], periodontal disease, and tooth decay [4] are among the most common oral diseases that present a huge health problem.

Although significant advances in understanding the cellular and molecular events of oral diseases have been made, only a few approved therapies are available at present [3]. This is because the environmental conditions in the oral cavity, such as saliva-washing effects associated with accidental swallowing or enzymatic degradation of the drug, limit the usefulness of existing medications such as mouthwashes, gels, solutions, and oral suspensions [5]. Hence, there is a need for improvements of treatment strategies that will increase the efficiency of oral therapy. One highly promising technological approach in this rapidly emerging field is the design of nanocarriers [6]. Such systems can be transferred into films or gels [7, 8] that can be applied to the lining mucosa, e.g., to the inner side of the cheek. The lining mucosa represents the larger part of the oral cavity and allows better absorption of drugs than the keratinized mucosa. Thereby, transport of nanoparticles (NPs) occurs either via the transcellular or the paracellular pathway [9, 10].

However, during medical application, NPs encounter saliva as the first protective biological barrier. Saliva is a hypotonic fluid with low ionic strength that contains calcium, phosphate, carbonate, and thiocyanate ions [11]. Various proteins such as MUC7, secretory IgA, and lactoferrin are present in saliva, constituting the salivary immune defense system that promotes the clearance of xenobiotics due to agglomeration effects [12, 13]. Moreover, the high-molecular-weight mucin MUC5B forms an entangled mucus network [14–16] due to different intermolecular interactions, including calcium-mediated cross-links, hydrophobic interactions, and carbohydrate–carbohydrate interactions [17, 18]. This network is responsible for the gel-like structure and the viscoelastic properties of saliva, since the linked mucin fibers (elastic component) possess a high water binding capacity (viscous component) [16, 19]. These mucins, together with the membrane-associated epithelial MUC1, as well as sIgA, and cystatin adsorb on the epithelial cell surface by hydrophobic interactions, which was previously shown by Gibbins et al. [20, 21]. Thereby, the bound mucosal pellicle is formed, which is a supra-molecular film that functions as lubricant with high moisture retention capacity.

Although the impact of physicochemical properties of NPs on colloidal stability in artificial media simulating, e.g., salivary pH and ionic conditions and buccal cell uptake using serum-free culture medium or phosphate buffer has been demonstrated [22, 23], to the best of the authors’ knowledge, no systematic investigation of well-defined NPs (i.e., NPs of nominal size, surface charge, and hydrophilicity) dispersed in relevant media including unstimulated whole-mouth saliva (UWMS) has been performed. Thus, it still remains unclear whether NPs interact with components of UWMS, and how size and surface functionalization (charge) impact the colloidal stability and, consequently, the mobility of NPs as a function of the salivary microstructure. NP agglomeration, because of interactions with, e.g., salivary proteins and ions, would implicate a significant increase in size. This would either suppress diffusion of particles larger than the salivary mesh size or lead to immobilization because of interaction filtering. In both cases, this would impede cellular interactions and, concurrently, the transport of active drug candidates to the site of action. Thus, by successfully addressing saliva as the first protective barrier, the most important nanocarrier design features can be assessed and useful information regarding the relationship between the physiological liquid and NPs considering physicochemical properties will help to improve treatment strategies in oral diseases.

To address this issue, we first investigated the agglomeration tendency and then the mobility of 20- and 200-nm (i) non-functionalized, (ii) carboxylated, and (iii) aminated polystyrene model NPs as a function of salivary microstructure and composition. Finally, cellular uptake was studied with a focus on particles with a nominal diameter of 200 nm, as we have recently demonstrated that this is an optimal size for efficient buccal uptake [23].

## Materials and methods

### Saliva collection

UWMS was collected from six healthy volunteers, aged between 25 and 45 years, who refrained from eating, drinking, and mouth-cleaning products 2.0 h prior to saliva collection. UWMS was drooled into tubes until 2.0 ml had been collected. The samples were characterized with respect to the whole protein concentration, osmolality, and pH to ensure comparability of the samples (see also Supplementary Material).

### NP characterization studies

Fluorescence-labeled non-functionalized 20- and 200-nm (red, Thermo Scientific), carboxylated 20- and 200-nm (red, Molecular Probes), and aminated 20-nm (yellow, Merk) and 200-nm (red, Molecular Probes) polystyrene NPs were used to model NPs with neutral, negative, and positive surfaces. Non-functionalized polystyrene NPs are slightly negative, which is a result of remaining fragments of the initiator used to start the polymerization reaction [24]. However, these particles are considered to be plain or neutral, because they are not specifically functionalized. The model NPs were dispersed in UWMS, salivary buffer (SAGF) [11], and Milli-Q (MQ) water. SAGF was prepared as previously described and consisted of mono- and multivalent ions [25]. In brief, it was composed of NaCl (125.6 mg/l), KCl (963.9 mg/l), KSCN (189.2 mg/l), KH_2_PO_4_ (654.5 mg/l), urea (200 mg/l), Na_2_SO_4_∙10H_2_O (763.2 mg/l), NH_4_Cl (178 mg/l), CaCl_2_∙2H_2_O (227.8 mg/l), and NaHCO_3_ (630.8 mg/l) dissolved in MQ water. The ionic strength was 43 mM, and the pH was 6.8. To prevent the loss of CO_2_ gas, the buffer was freshly prepared prior to use. To compare the influence of monovalent ions, a 43 mM NaCl solution was prepared. The hydrodynamic particle size and the polydispersity index (PdI) were measured using a dynamic light scattering (DLS) instrument (Zetasizer Nano ZSP, Malvern), and the zeta potential was determined via laser Doppler velocimetry using a Zetasizer Nano ZSP (Malvern) as previously described [23].

### Studies of NP interactions with salivary mucoglycoproteins

To examine the surface-bound mucoglycoproteins, NPs were incubated with UWMS (100 μg/ml) for 30 min (*n* = 2) and centrifuged to pellet the NP–saliva complexes (30 min at 14,000 rpm at 4 °C). The pellet was then resuspended in SAGF buffer and centrifuged again. This step was repeated three times. Proteins were eluted from the NPs by adding SDS–sample buffer (62.5 mM Tris–HCl, pH 6.8; 2% *w*/*v* SDS, 10% glycerol, 50 mM DTT, 0.01% *w*/*v* bromophenol blue) to the pellet and incubated at 95 °C for 5 min.

For 1D gel electrophoresis, 20 μl of the recovered NPs in SDS–sample buffer was separated on a 12% SDS–polyacrylamide gel. Pure saliva (total volume 20 μl, i.e., 2 μl saliva + 18 μl SDS–sample buffer) served as a control. The gels were run at a constant voltage of 200 V for 35 min and stained with Periodic acid Schiff (PAS) stain to detect mucins after the gel electrophoresis [26]. The gels were fixed in 50% methanol for 30 min and gently washed three times in 3% glacial acetic acid for 10 min. Oxidation was performed in 2% periodic acid for 15 min, and subsequently, the gels were gently washed three times in 3% glacial acetic acid for 5 min. Schiff’s reagent (VWR) was then added for 15 min in the dark under constant agitation to complete the PAS staining procedure. Immunoblotting was conducted as described previously [27]. The antibodies used were α-MUC5B (Abcam) and goat α-rabbit immunoglobulin G (IgG) Ab conjugated with horseradish peroxidase (Santa Cruz). Enhanced chemiluminescence (ECL) was used to detect the peroxidase activity. Horseradish peroxidase enzyme is tethered to the secondary antibody. The ECL substrate luminesces when exposed to the reporter on the secondary antibody. The light is then detected by a CCD camera which captures a digital image of the immune blot.

### Determination of the salivary network structure

The architecture of unstimulated human whole saliva was examined by the use of freeze fracture transmission electron microscopy (TEM). For this purpose, the samples were frozen in liquid propane, stored in liquid nitrogen, and fractured in a Balzers BAF400D freeze-etching apparatus under vacuum (at a pressure between 1.3 × 10^−4^ and 1.3 × 10^−5^ Pa). To produce replicas, vacuum was applied. The surface was sputtered with platinum and carbon, and the height was controlled with a quartz crystal thin-film monitor. The replicas were cleaned with a sodium hypochlorite solution for 3 h, stored in 50% NaOH, and washed three times with distilled water before being mounted on an uncoated copper grid. The samples were visualized using a model FEI-Tecnai-20 TEM instrument equipped with a camera (Gatan US1000) and operated at an acceleration voltage of 120 kV. The pore–size distribution of at least 500 pores was calculated from the TEM images using the ImageJ-Fiji software package. For this purpose, the images were converted into binary files, and the Feret diameters of the white areas were calculated.

### Rheological investigations of saliva

Microrheological experiments were performed using an inverted Nikon Eclipse Ti microscope equipped with laser tweezers (Tweez 250si, Aresis). The focused beam contained a strong electric field gradient, and thus, dielectric particles could be manipulated by means of entrapment in the beam waist. The infrared laser beam, with a wavelength of 1064 nm, was focused through a water immersion objective (Nikon, ×60, numerical aperture 1.00) in a sample cell and was used to trap and manipulate silica beads (SS04N, Bangs Labs, diameter of 2.32 μm). One microliter of a silica bead/MQ water solution (0.1 μg/ml) was added to 50 μl human whole saliva and pipetted into a sample chamber formed from two coverslips separated by spacers of approximately 200 μm in thickness. The sample chamber was sealed with silicone glue to prevent evaporation. To eliminate surface effects from the measurement, the trapping plane was set at least 20 μm from the walls of the sample cell. The position of the optical trap was sinusoidally modulated with a constant amplitude of 0.3 μm and frequencies of 25–0.2 Hz. The bead was trapped at the periphery of the salivary mucin network, and the position was recorded using a CMOS camera (UI-3370CP-M-GL) at 200 frames per second. The image acquisition performed by the camera was synchronized with the trap movement using an external camera trigger to ensure that the phase lag between the bead and the trap position could be exactly determined. The bead trajectories were obtained by analyzing the recorded videos using particle tracking software (PartTrack V3. 36 for Aresis Tweez). The bead and laser trap trajectories were further analyzed using custom analysis software written in MATLAB to obtain the microrheological parameters of each sample. The measurements were performed at room temperature (RT, 23 ± 2 °C), with no special control over the chamber temperature. Gravity effects were not detected during calibration in water and, thus, not taken into account for data analysis. Prior to each measurement, the stiffness of the optical trap was recalibrated. The calibration method used was based on a statistical analysis of bead motion in a stationary trap. At least 30,000 frames were recorded during each calibration run. The trap stiffness was determined by analyzing the distribution of the bead positions using the TweezPal software.

Macrorheological measurements were conducted using a Physica MCR 301 rheometer (Anton Paar) in a cone–plate geometry (CP50-1) at 24 °C under the application of strain-controlled oscillation. To prevent water evaporation and the adsorption of protein molecules at the periphery of the geometry, a small amount of 0.1% sodium dodecyl sulfate (SDS, Sigma-Aldrich) was applied around the rim of the geometry, as described previously [28]. The linear viscoelastic region was defined using an amplitude sweep, and a strain of 5% was chosen for the subsequent oscillation measurements to ensure minimal shearing damage. The storage modulus (*G*′), the loss modulus (*G*″), and the complex viscosity were extracted from frequency sweep tests with deformations between 10 and 0.1 Hz.

### NP diffusion velocity measurements

To evaluate the impact of surface charge on NP behavior in saliva, the diffusion velocity was recorded using NP Tracking Analysis (NTA, NanoSight LM10, Malvern) at RT (23 ± 2 °C) with a green laser (532 nm) and then with a blue laser (488 nm). The technology of NTA is based on the principles of the DLS of NPs dispersed in a transparent medium. Since saliva is a complex fluid that also contains particulate matter, fluorescence measurements were performed with fluorescent-labeled polystyrene NPs using a 565-nm long-pass filter for red fluorescence and a 500-nm long-pass filter for yellow fluorescence. Furthermore, the movement of 200-nm NPs in MQ water was recorded; however, the diffusivity of 20-nm NPs in water was too high for trajectory studies. The NP movement was recorded for 4 s using a high-sensitivity camera (Marlin), and the NP trajectories were analyzed using the ImageJ-Fiji software package with the TrackMate plugin. The one-dimensional mean-squared displacement (MSD) was calculated from the *x* coordinates of the NP trajectories as follows (Eq. 1):

1 $$ \mathrm{MSD}\left(\tau \right)={\left[ x\left( t+\tau \right)- x\left(\tau \right)\right]}^2 $$

where *x* is the position of the NP at time *t* and *τ* is the lag time.

The effective diffusivity (*D*_eff_) was calculated as follows (Eq. 2):

2 $$ {D}_{\mathrm{eff}}=\frac{\mathrm{MSD}}{2\tau} $$

The theoretical diffusion coefficients (*D*_0_) for 20- and 200-nm NPs in water and saliva were calculated using the Stokes–Einstein equation (Eq. 3).

3 $$ {D}_0=\frac{k_B T}{3\pi \eta d} $$

where *k*_*B*_ is the Boltzmann constant, *T* is the temperature (i.e., 298.15 K), *η* is the viscosity of the fluid, which has a value of 8.91 × 10^−4^ Pa s for water and was approximated as 3 × 10^−3^ Pa s for saliva based on the microrheology data, and *d* is the NP diameter.

### NP uptake studies

To determine whether NPs dispersed in saliva remain small enough to interact with epithelial cells, the human buccal non-keratinized TR146 cell line (Sigma-Aldrich) was used and incubated with particles dispersed in saliva. For confocal laser scanning microscopy (cLSM) studies, 1.5 × 10^4^ cells/cm^2^ were seeded into eight-well culture slides (BD Falcon) and incubated for 7 days under standard conditions at 37 °C in a humidified 5% CO_2_ atmosphere. NPs dispersed in UWMS and 1% PenStrep (100 μg/ml) were applied on the cell monolayers and incubated for 1 min and for 1 h with a maintained cell viability of >80%, which was evaluated prior to the experiments (MTS test, Promega) [23]. The cell nuclei were stained with Hoechst 33,342 (Invitrogen), and the cytoskeletons of the cells were stained with tetramethylrhodamine (TRITC)-labeled phalloidin (Invitrogen) or fluorescein isothiocyanate (FITC)-labeled phalloidin (Sigma-Aldrich) according to the manufacturer’s instructions. The cells were fixed with a 4% formalin solution (Sigma-Aldrich), and cLSM studies were performed [29] using a Zeiss LSM 510 META microscope equipped with the ZEN software package. To quantify the uptake of NPs into buccal cells, TR146 cells were seeded into 24-well plates (1.5 × 10^4^ cells/cm^2^) and cultured for 7 days. The medium was then replaced with 100 μg/ml NP/saliva suspensions and incubated for 1 min and 1 h. Subsequently, the cells were washed twice with HBSS (Invitrogen) and lysed with a 2% Triton X-100/MQ water solution (Sigma-Aldrich) for 30 min. The fluorescence intensity was measured using a spectro-fluorimeter (FLUOStar Optima, BMG, Labortechnik) at 544/590 nm for red fluorescence and 485/520 for yellow fluorescence.

## Results

### Particle agglomeration in saliva and physiological buffer systems

Particle characterization studies were performed in MQ water, SAGF, saliva, and 43 mM NaCl using DLS. The results are summarized in Table 1. The analyses showed that in MQ water, smallest sizes were recorded. In saliva, all investigated NPs agglomerated to a certain extent (see also Fig. S1 of the Supplementary Material). A significant increase in NP size was observed for all 20-nm NPs (≥100 nm) and for 200-nm aminated NPs (>1000 nm) and carboxylated NPs (>2000 nm). By contrast, non-functionalized 200-nm NPs were only moderately affected (the mean diameter increased to approximately 360 nm).

**Table 1:** NP characterization studies in physiologically relevant media

NP type	Size (nm)	PdI	*ζ* (mV)	Size (nm)	PdI	*ζ* (mV)	Size (nm)	PdI	*ζ* (mV)	Size (nm)	PdI	*ζ* (mV)
	MQ water			SAGF			Saliva			NaCl		
200-nm aminated	298 ± 5	0.176 ± 0.020	53 ± 2	1474 ± 133	0.339 ± 0.059	4 ± 1	1083 ± 185	0.246 ± 0.032	−18 ± 2	284 ± 4	0.142 ± 0.013	19 ± 1
200-nm carboxylated	261 ± 2	0.103 ± 0.021	−52 ± 1	390 ± 2	0.196 ± 0.010	−62 ± 14	2347 ± 193	0.185 ± 0.035	−22 ± 1	342 ± 7	0.314 ± 0.069	−42 ± 3
200-nm non-functionalized	238 ± 4	0.105 ± 0.047	−47 ± 2	226 ± 1	0.143 ± 0.006	−47 ± 2	361 ± 16	0.116 ± 0.013	−22 ± 2	245 ± 3	0.194 ± 0.014	−44 ± 3
20-nm aminated	21 ± 6^a^	0.265 ± 0.014	−36 ± 2	20 ± 1^a^	0.207 ± 0.028	−23 ± 6	142 ± 5	0.410 ± 0.024	−19 ± 2	27 ± 7^a^	0.206 ± 0.117	−21 ± 1
20-nm carboxylated	53 ± 1	0.260 ± 0.006	−56 ± 2	51 ± 5	0.232 ± 0.092	−32 ± 2	99 ± 2	0.309 ± 0.031	−17 ± 0.3	45^b^	0.273 ± 0.011	−39 ± 4
20-nm non-functionalized	22^b^	0.139 ± 0.015	−42 ± 1	22^b^	0.144 ± 0.013	−46 ± 14	138 ± 2	0.319 ± 0.040	−19 ± 0.3	22^b^	0.177 ± 0.011	−32 ± 1

The hydrodynamic diameters, PdI values, and zeta potential values of aminated, carboxylated, and non-functionalized NPs dispersed in MQ water, SAGF buffer, human whole saliva, and 43 mM NaCl solution are shown. The values are presented as the mean ± SD results from three independent experiments

^a^The multimodal size distribution was measured, and the predominant size fraction (>75%) is represented

^b^The SD is lower than the limit of quantification

To assess whether agglomeration is triggered by mono- and/or multivalent salivary ions, salivary buffer (i.e., SAGF) and 43 mM NaCl was used. Salivary ions were found to influence neither 200-nm non-functionalized NPs nor 20-nm NPs, as the observed NP sizes were comparable to those observed in water. However, 200-nm aminated NPs agglomerated in SAGF to a high extent (1474 ± 133 nm); in 43 mM NaCl, no agglomeration was observed and hydrodynamic diameters and PdIs were comparable to those obtained in water (284 ± 4 versus 298 ± 5 nm). Two hundred-nanometer carboxylated NPs showed moderate agglomeration in both SAGF and NaCl buffers, and the hydrodynamic diameters ranged from 390 ± 2 to 342 ± 7 nm.

### Particle interactions with salivary proteins

**Fig. 1:**
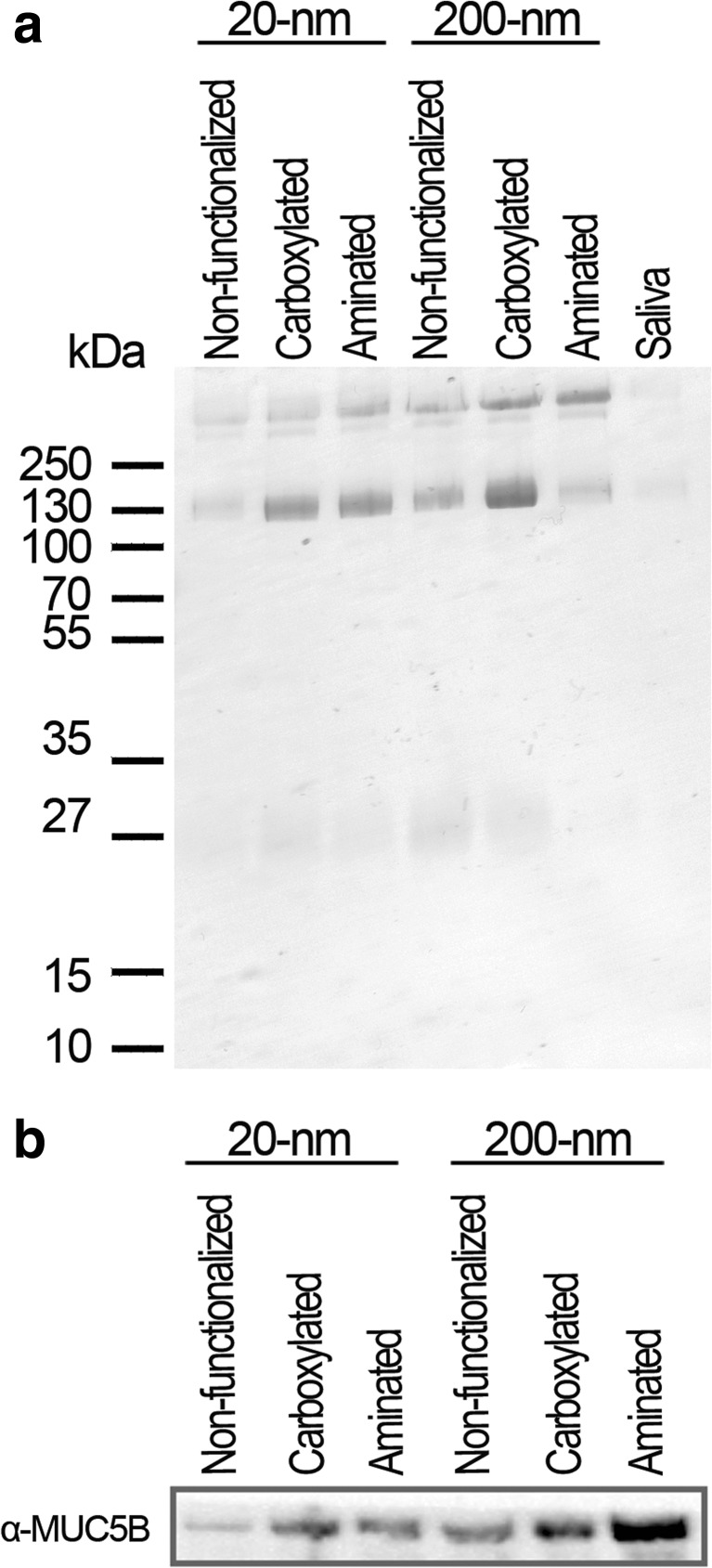
Identification of the interactions of NPs with salivary mucoglycoproteins. The NPs were incubated in saliva for 30 min at RT and separated from unbound salivary components by centrifugation. **a** PAS staining was performed after 1D gel electrophoreses to determine the surface bound mucins. The band at 130 kDa is distinctive for MUC7, indicating that this mucin is highly abundant at the surface of carboxylated NPs. **b** To identify the presence of the high molecular mucin MUC5B, western blotting was conducted. MUC5B was found to bind to all particle surfaces, but to a greater extent to carboxylated and aminated NPs. The experiments were replicated two times

To determine if salivary proteins bind to the NP’s surface, 1D gel electrophoresis and immunoblotting were performed. We specifically focused on the identification of the two predominant salivary mucins MUC7 and MUC5B. MUC7, which has a characteristic band at 130 kDa [20], bound to all particle surfaces but was especially abundant on the surface of carboxylated NPs (Fig. 1a). MUC5B weakly bound to non-functionalized NPs and was more abundant on the surface of functionalized NPs (Fig. 1b). Overall, mucins were more dominant at the surface of 200-nm NPs compared to 20-nm NPs.

### Biophysical characterization of human saliva

**Fig. 2:**
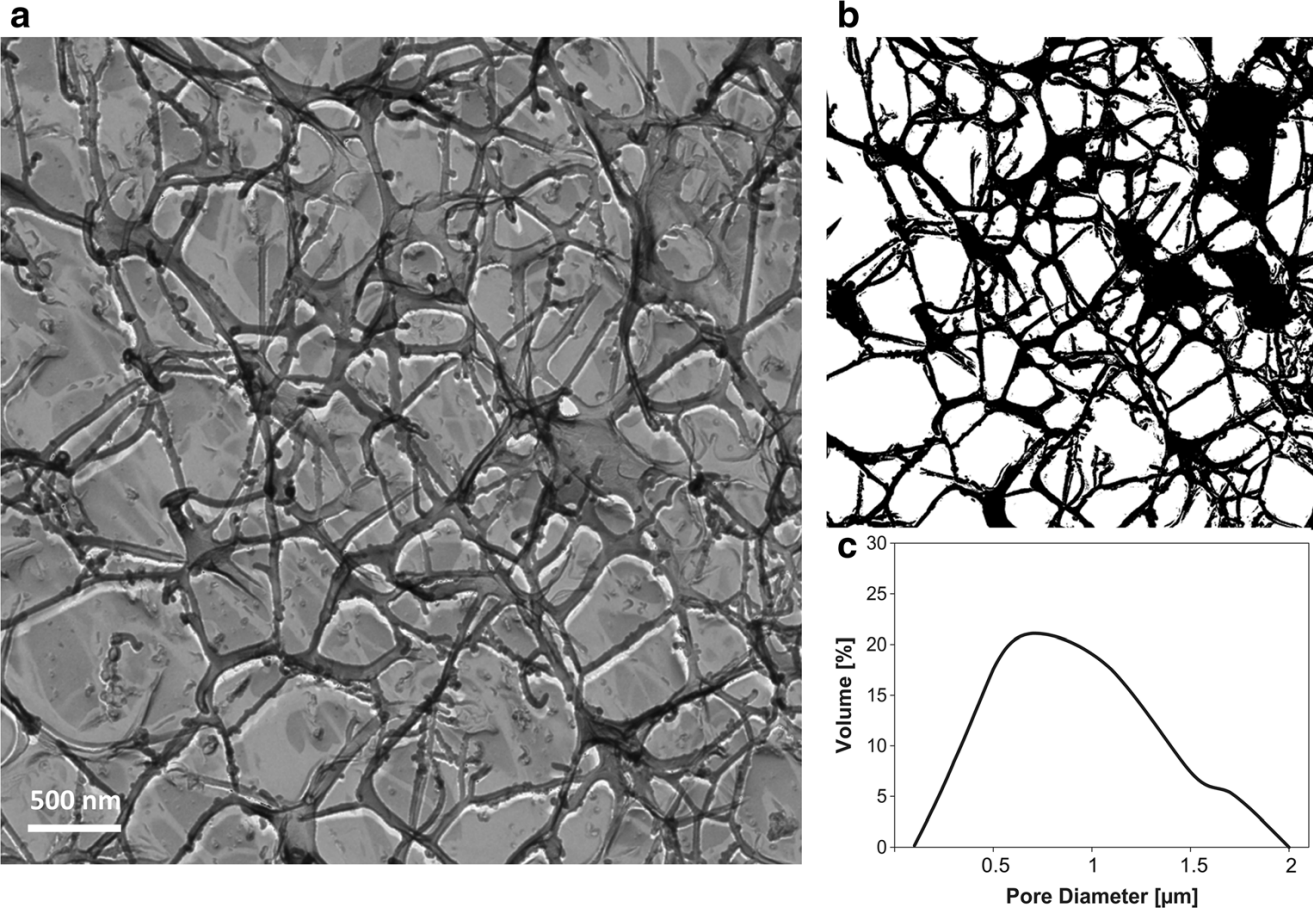
Analysis of the salivary microstructure. **a** A representative TEM image of freeze fracture replicas shows the architecture of the salivary mucus network. **b** The 2D image in **a** was also converted into a binary file. **c** The Feret diameters of at least 500 pores were calculated using the ImageJ software package. The pore–size distribution (presented as volume distribution Q3) is broad, ranging from 100 to 2000 nm

We investigated saliva in its native state by using freeze etching combined with TEM. It was found that the mucin fibers form a coherent mucin network (Fig. 2a) but are also arranged individually. The pore–size distribution of the salivary mucin network was analyzed from the 2D TEM images (Fig. 2b), and the Feret diameters were calculated (Fig. 2c). The mode diameter of the mesh pores was determined to be 0.7 μm, with a peak width of 1.9 μm.

**Fig. 3:**
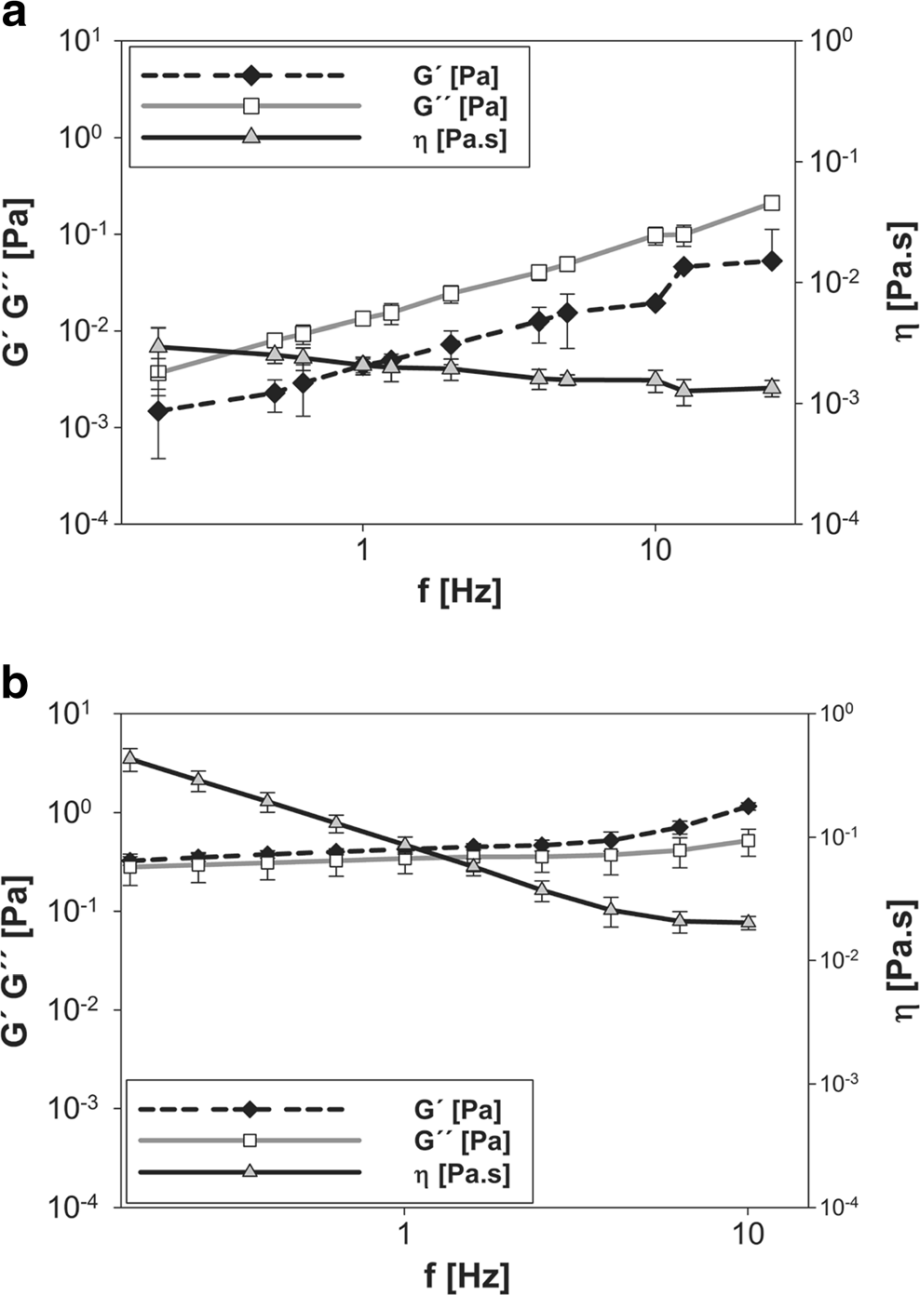
Rheological analysis of human whole saliva. **a** Microrheology was measured using optical tweezers to study the viscoelastic properties of the fluid inside the pores. *G*″, the viscous modulus, is markedly larger than *G*′, the elastic modulus, indicating a non-associated system with a viscosity *η* ranging from 3.0 × 10^−3^ to 1.3 × 10^−3^ Pa s. **b** Macrorheological measurements were conducted using a strain-controlled rheometer in a cone–plate geometry. *G*′, the elastic modulus is markedly larger than *G*″, the viscous modulus, and the viscosity *η* is dependent on the applied frequency, indicating a viscoelastic behavior. The values are presented as the mean ± SD results from three independent experiments

Furthermore, the viscosity of the fluid of the surrounding and the pores of the network were measured. While bulk rheological techniques such as the cone–plate rheometer take into account the viscoelastic behavior of the whole fluid including the elastic response of the mucin fibers (i.e., macrorheology), optical tweezers are used to study the fluid’s rheology at the microscale (i.e., microrheology). A probe particle was trapped at the periphery of the mucin network, and shear stress was induced by modulating its position at various frequencies with a sinusoidal pattern. The response of the fluid resulted in a relative phase lag and reduction in amplitude of the particle’s movement. From those data, the viscoelastic parameters were calculated. Our data demonstrate that the microrheology of saliva (Fig. 3a) is significantly different from its viscoelastic macrorheology (Fig. 3b). The loss modulus (*G*″), which is a measure of the viscous component, is markedly larger than the storage modulus (*G*′), which represents the elastic component, indicating a weakly structured fluid. Moreover, the viscosity ranges from 3.0 × 10^−3^ to 1.3 × 10^−3^ Pa s at RT (depending on the applied shear stress), showing that the pores of the salivary mucus network are filled with a fluid of a viscosity slightly higher than that of water.

### Particle mobility in saliva

**Fig. 4:**
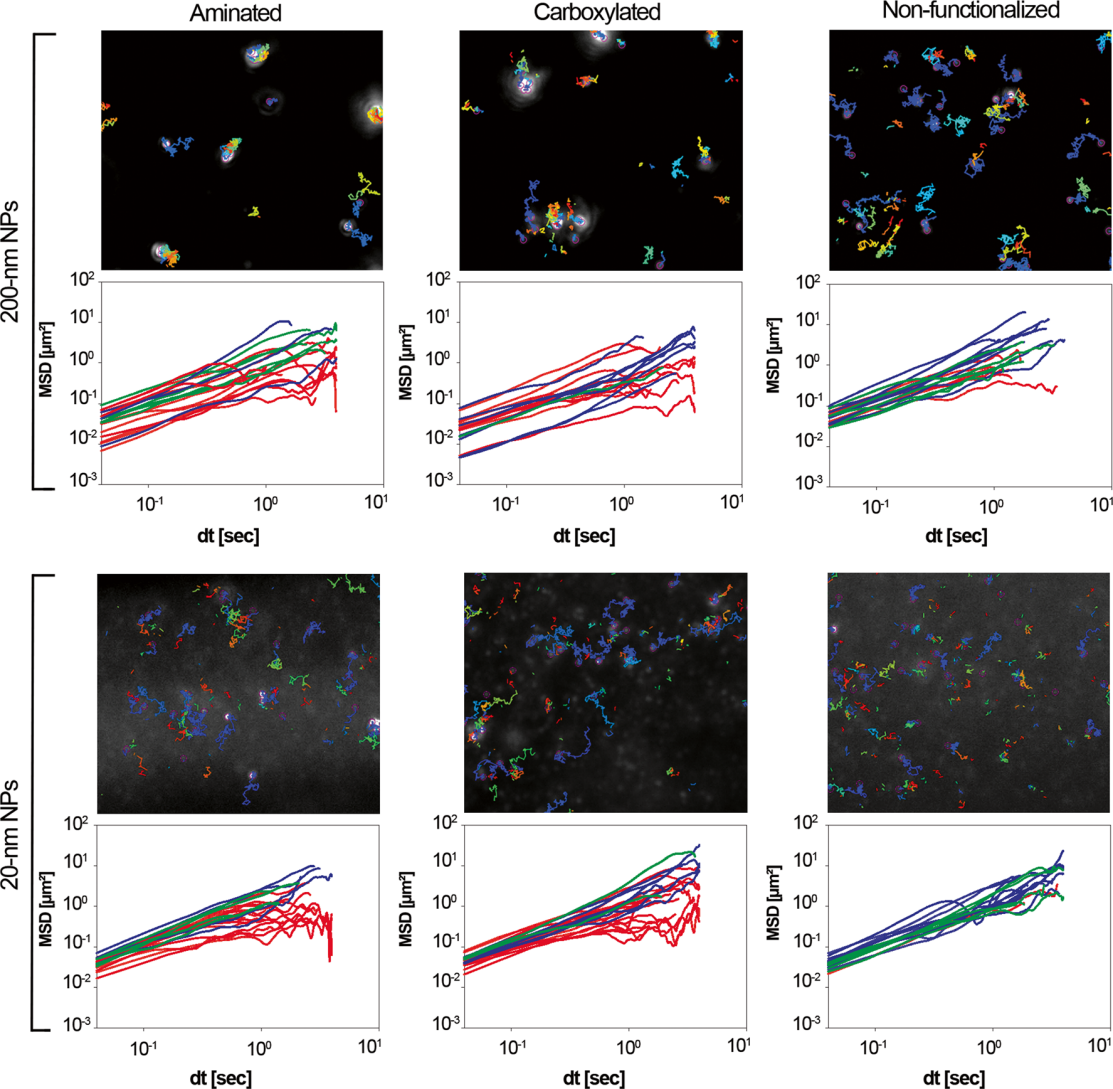
NP trajectory studies in saliva. The diffusion of the NPs was recorded by means of NTA. The *upper panels* show representative images of NP trajectories, and the *lower panels* depict the corresponding MSD plots of individual NPs (*n* ≥ 20) for up to 4 s. The colored MSD curves represent normal diffusion (*green*, *α* = 1), sub-diffusion (*red*, *α* < 1), and super-diffusion (*blu*e, *α* > 1). The 200-nm aminated and carboxylated NPs showed the slowest motion, and their MSD curves reveal a broad distribution range. The 200-nm non-functionalized NPs were more diffusive, with an average MSD value of more than twofold higher at a timescale of 0.5 s. At short timescales, the 20-nm NPs showed interchangeable diffusion patterns. However, at longer timescales, the motion of the 20-nm functionalized NPs was hindered, and thus, a large number of sub-diffusive tracks are present. By contrast, the diffusion patterns of the 20-nm non-functionalized NPs reflect predominantly normal and super-diffusion behaviors

To determine the NP dynamics in saliva, NP tracking studies were performed. We quantified the NP trajectories by calculating the MSD (Fig. 4). In general, the diffusion of the NPs was slower in saliva than in water (see Table 2 and Fig. S2 of the Supplementary Material). In saliva, a higher diffusivity was observed for 20-nm NPs than for 200-nm aminated and carboxylated NPs (Fig. 4). However, there is no significant difference between the *D*_eff_ of 200-nm non-functionalized NPs and 20-nm NPs at a timescale of 0.52 s (*P* > 0.05 according to Student’s *t* test).

**Table 2:** Theoretical diffusion coefficients (*D*_0_) and effective diffusivities (*D*_eff_) of NPs in water and saliva

NP type (nm)	*D*_0_ (μm^2^/s) water	*D*_eff_ (μm^2^/s) water	*D*_0_ (μm^2^/s) saliva	*D*_eff_ (μm^2^/s) saliva	
200	2.45	2.08 ± 0.90	0.73	Aminated	0.38 ± 0.25
				Carboxylated	0.30 ± 0.34
				Non-functionalized	0.60 ± 0.32
20	24.51	n.d.	7.28	Aminated	0.63 ± 0.37
				Carboxylated	0.58 ± 0.31
				Non-functionalized	0.62 ± 0.30

*D*_0_ was calculated at a temperature of 25 °C, and the viscosity was approximated as 3 × 10^−3^ Pa s for saliva based on the microrheological studies. *D*_eff_ values were calculated at a lag time of 0.52 s

*n.d.* not determinable

The diffusion patterns of the NPs were also characterized in terms of the slope (*α*) of the logarithmic plot of the MSD versus the timescale: *α* = 1 indicates unobstructed Brownian diffusion, *α* < 1 indicates hindered diffusivity, also referred to as sub-diffusion, and *α* > 1 indicates super-diffusion [30]. The diffusion patterns of the non-functionalized NPs were mostly unobstructed or even super-diffusive because of collisions between these highly diffusive NPs (*D*_eff_ = 0.6 μm^2^/s). The functionalized NPs exhibited predominantly obstructed diffusion with *α* < 1, independent of the NP size.

### Particle uptake into oral cells

**Fig. 5:**
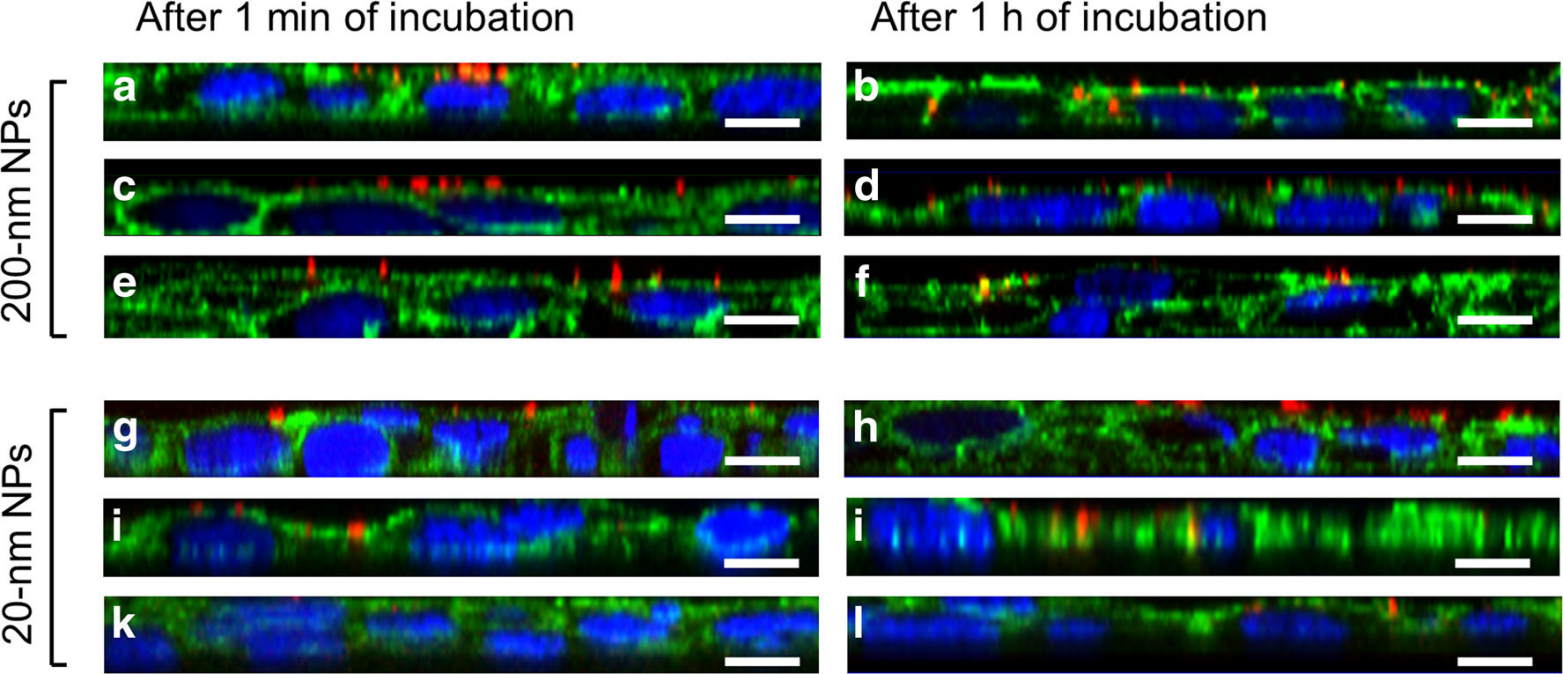
Salivary permeation studies. (Non-)functionalized NPs (*red*) were dispersed in saliva and exposed to a confluent TR146 cell layer, and virtual z stacks were acquired via cLSM. **a** The orthogonal view illustrates that 200-nm non-functionalized NPs efficiently permeated the saliva within 1 min and reached the cell membrane (*green*). **b** After 1 h of incubation, 2.8 ± 0.4% of the NPs were internalized by the buccal cells. Similarly, 200-nm **c** carboxylated and **e** aminated NPs also passed through the salivary barrier within 1 min and reached the oral cells. However, 200-nm **d** carboxylated and **f** aminated NPs were not taken up by the TR146 cells after 1 h (≤0.5% uptake rate) due to the large agglomerate size. Only a small amount of 20-nm **g**, **h** non-functionalized, **i**, **j** carboxylated, and **k**, **l** aminated NPs were found at the cell membrane after 1 min and 1 h of incubation, respectively. The cell nuclei are depicted in *blue*, and the *scale bars* represent 10 μm

NPs were dispersed in saliva and incubated with a non-keratinized buccal TR146 cell line. The results showed that the 200-nm non-functionalized NPs were immediately (after 1 min) detected close to the cell membrane (Fig. 5a). After 1 h, 2.8 ± 0.4% (*w*/*v*) of the NPs were internalized by the cells (Fig. 5b). Furthermore, 200-nm aminated (Fig. 5c, d) and carboxylated NPs (Fig. 5e, f) were also found at the cell membrane, but cellular uptake was inhibited (uptake rate ≤0.5% (*w*/*v*) after 1-h incubation time). Notably, the uptake capacity of 20-nm NPs was low, with an uptake rate of ≤1.0% (*w*/*v*) (Fig. 5g–l).

## Discussion

**Fig. 6:**
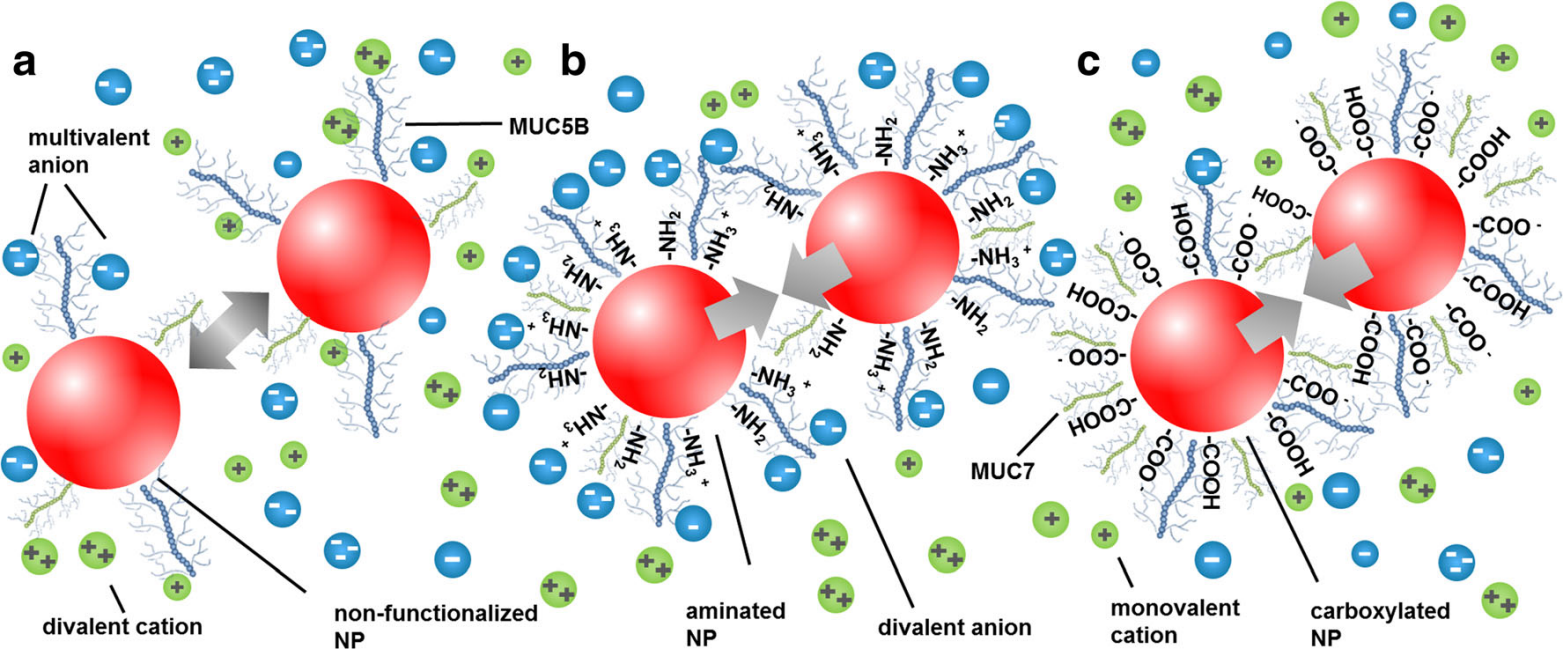
Schematic illustrations of the agglomeration behaviors of 200-nm NPs in saliva. **a** Non-functionalized NPs are rather stable in saliva and show a low binding affinity to the main salivary mucins MUC7 and MUC5B, while the surface charge of **b** aminated NPs is screened by multivalent anions, which leads to agglomeration. Moreover, aminated NPs bind to MUC5B, which is also abundant on the surface of **c** carboxylated NPs. However, they favor the binding of MUC7, which results in destabilization and agglomeration

Saliva, which is a highly complex fluid composed of water, proteins, enzymes, and ions [13], consists of pores ranging from the micro- to the nanoscale (Fig. 2). This corroborates that the salivary microstructure enables diffusion of NPs and, thus, adsorption on the epithelial mucosa. Furthermore, it was found that the pores of the salivary network are filled with a low-viscosity fluid that does not impact the mobility of NPs (Fig. 3). However, surface functionalization and size were found to critically affect the NP behavior (Table 1). Compared to non-functionalized particles (Fig. 6a), 200-nm aminated NPs significantly agglomerated in saliva. Studies performed with SAGF showed that this was triggered by multivalent anions, such as PO_4_^3−^, SO_4_^2−^, and CO_3_^2−^. They rapidly interacted with the amino groups and screened the positively charged surface (zeta potential of +4 mV; Table 1, Fig. 6b), which led to agglomeration (>1000 nm). Additional studies performed with NaCl buffer, which only consisted of dissolved monovalent ions, showed no impact on the hydrodynamic diameter, and the zeta potential of the NPs was still 19 ± 1 mV, indicating high physical stability. These results are in accordance with studies by Ngyen et al. and Quesada–Perez and coworkers [31, 32]. They showed that this colloidal instability cannot be explained by the classical Derjaguin–Landau–Verwey–Overbeek (DLVO) theory alone, because once ions with a higher valence are available, ion–ion interactions must be considered. When multivalent counterions adsorb on a NP surface, strong interactions develop, which cause additional ion attraction and, thus, screening of the surface charge.

The 200-nm carboxylated NPs were only moderately affected by ions contained in SAGF and NaCl buffers. Thus, the interactions of mono- and multivalent salivary ions with the negatively charged carboxyl groups are negligible, presumably because of the presence of predominantly monovalent cations (i.e., Na^+^ and K^+^) and only a small amount of divalent cations (i.e., Ca^2+^). This indicates that agglomeration is mainly triggered by the salivary proteins (Fig. 6c).

Notably, surface functionalization played a minor role in the agglomeration behavior of 20-nm NPs. Similar agglomerate sizes were obtained for functionalized and non-functionalized NPs, ranging from 99 to 142 nm (Table 1). Moreover, compared with 200-nm functionalized NPs, 20-nm NPs were not affected by multivalent salivary ions. We anticipate that the high NP surface curvature of 20-nm NPs reduces the binding efficiency of multivalent ions because of a higher steric hindrance, which was also described for BaTiO_3_ particles [33]. Thus, 20-nm NPs favor the adsorption of monovalent ions, indicating that ion–ion interactions and the related charge screening effects can be neglected.

Interactions with mucins might impact not only agglomeration but also NP diffusion; thus, NP tracking studies were performed. The theoretical diffusion coefficient (*D*_0_) for non-functionalized 200-nm NPs was only slightly higher in saliva than the effective diffusivity (*D*_eff_) (Table 2). Due to their small sizes, they diffused in saliva without interacting. Notably, the mobility of 20-nm NPs and 200-nm functionalized NPs was significantly affected (Fig. 4). This can be attributed to their large agglomerate sizes, which reduced their effective diffusivity (*D*_eff_). Moreover, functionalized NPs showed a hindered diffusion pattern (*α* > 1), implying that they interact with the salivary mucus network and are increasingly immobilized during transit.

**Fig. 7:**
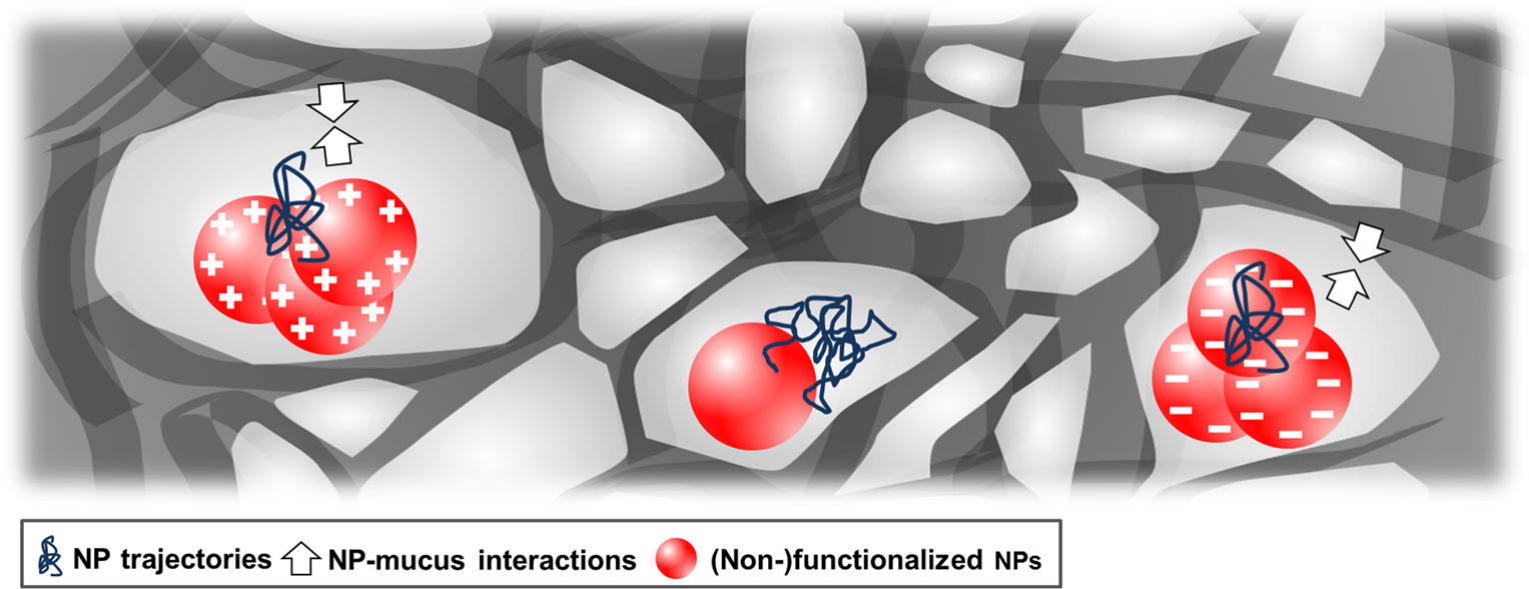
A schematic illustration of the effective motion of 200-nm NPs. Aminated and carboxylated NPs agglomerate in saliva and bind to MUC5B, the main component of the salivary mucus network, becoming effectively immobilized. Non-functionalized NPs are rather stable in saliva, and the mucin binding affinity is low, leading to an unhindered diffusivity

To assess whether this immobilization is caused due to interactions with MUC5B, the main component of the salivary mucus network, and/or MUC7, immunoblot analysis was performed (Fig. 1b) [27, 34]. The peptide chain of MUC5B carries heavily glycosylated hydrophilic domains in addition to less glycosylated regions, also known as hydrophobic patches [35]. It seems that non-functionalized NPs weakly bind to those patches, mainly driven by hydrophobic forces and/or van der Waals interactions [35, 36]. By contrast, functionalized NPs bind to MUC5B more strongly, most likely because of electrostatic interactions with negative and positive mucin domains, restricting their diffusivity (Fig. 7). MUC5B was found to be highly abundant on the surfaces of the 200-nm aminated NPs because, under physiological conditions, a net negative charge is prevalent as a result of sialic acid and sulfated sugar residues [37]. Interestingly, these results are contradictory to Gibbins and coworkers [21]. They investigated interactions of differently charged microparticles with the mucosal pellicle and found that only hydrophobic particles bound to MUC5B at a very low level. However, in the soluble phase of UWMS, MUC5B may become more charged associated with increased binding levels [16, 38]. Thus, together with the higher surface reactivity of NPs, interactions between functionalized NPs and MUC5B increase, resulting in higher binding efficacy. Moreover, the binding efficacy of MUC5B was lower for 20-nm NPs than for 200-nm NPs, because steric hindrance impedes the absorption of large proteins on NPs with a high surface curvature [39, 40].

MUC7 consists of a single glycosylated region and is surrounded by small non-glycosylated domains under physiological conditions showing an overall negative charge [41]. We showed that MUC7 was abundant on the surfaces of the carboxylated NPs (Fig. 1a). Thus, we conjecture that MUC7 triggers the agglomeration of carboxylated NPs due to van der Waals interactions. Our data coincide with data obtained by Gibbins et al. which showed that MUC7 also bound to negatively charged silica surfaces [21].

Recently, we showed that functionalization of NPs impacts cellular NP uptake. Positively and uncharged particles dispersed in serum-free cell culture medium showed higher cellular uptake rates compared to their negatively charged counterparts [23]. To evaluate whether the NPs, once in contact with saliva, also interact with the cellular site, time-dependent cLSM studies were performed with the human oral TR146 cell line (Fig. 5). As expected, 200-nm functionalized NPs were able to freely diffuse in saliva, were adsorbed on the cell surface, and were internalized by the epithelial cells (Fig. 5a, b). However, the uptake capacity was twofold lower than in serum-free cell culture medium [23]. Functionalized 200-nm NPs were found close to the cell membrane, most likely because of the sedimentation [42] of large agglomerates (≥1000 nm). Cellular uptake was inhibited due to the large agglomerate sizes (Fig. 5c–f). Although the 20-nm NPs were rather diffusive in saliva (*D*_eff_ values were comparable to that of the 200-nm non-functionalized NPs), the cellular uptake rate was low, indicating that the driving force was not sufficiently high to initiate wrapping of the cell membrane [23, 43] (Fig. 5g–l). This can be attributed to the specialized structure of the epithelial surface membrane, which is covered with ridge-like folds (i.e., microplicae), as we have recently shown [23].

## Conclusion

Within the limitations of the present study, such as the usage of an immortalized cancer cell line that only represents non-keratinized parts of the oral cavity, we demonstrated that the size and surface functionalization of NPs significantly modulate the interactions with saliva, more specifically their colloidal stability and mobility. Functionalized particles interacted with the salivary components to a greater extent than their uncharged counterparts, and NP size strongly impacted the uptake into TR146 cells. Thus, translating this knowledge into pharmaceutical formulation and focusing on application forms that do not require to remove saliva from the mucus surface or liquefy saliva temporarily mean that 200 nm non-functionalized NPs act as suitable carriers to deliver innovative drug candidates to the non-keratinized mucosa. Moreover, this work provides useful information regarding the relationship between biological barriers and NPs, which will help to properly discuss possible changes under inflamed conditions in future studies in order to improve treatment strategies [44, 45].

## Electronic supplementary material

ESM 1 (DOCX 1552 kb)
