# Supplementary material for: The effect of saliva on the fate of nanoparticles
Source: Clin Oral Investig. 2017 Jul 9;22(2):929–40. doi: 10.1007/s00784-017-2172-5 (PMC5820401; doi:10.1007/s00784-017-2172-5)
Supplement: Supplementary file 1 — (DOCX 1552 kb) [file 784_2017_2172_MOESM1_ESM.docx]

Supplementary Material: The effect of saliva on the fate of nanoparticles

Birgit J. Teubl ^1,2^, Biljana Stojkovic ^3^, Dominic Docter ^4^, Elisabeth Pritz ^5^, Gerd Leitinger ^1,5^, Igor Poberaj ^3^, Ruth Prassl ^2,6^, Roland H. Stauber ^4^, Eleonore Fröhlich ^2,7^, Johannes G. Khinast ^2,8,9^, Eva Roblegg ^1,2,9*^

^1^University of Graz, Institute of Pharmaceutical Sciences, Department of Pharmaceutical Technology and Biopharmacy, 8010 Graz, Austria

^2^BioTechMed, 8010 Graz, Austria

^3^University of Ljubljana, Faculty of Mathematics and Physics, 1000 Ljubljana, Slovenia

^4^Department of Nanobiomedicine/Mainz University Medical Center, 55131 Mainz, Germany

^5^Medical University of Graz, Institute of Cell Biology, Histology and Embryology, Research Unit Electron Microscopic Techniques, 8010 Graz, Austria

^6^Medical University of Graz, Institute of Biophysics, 8010 Graz, Austria

^7^Medical University of Graz, Center for Medical Research, 8010 Graz, Austria

^8^Graz University of Technology, Institute for Process and Particle Engineering, 8010 Graz, Austria

^9^Research Center Pharmaceutical Engineering, 8010 Graz, Austria

Corresponding Author:

Assoc.-Prof. Dr. Eva Roblegg

University of Graz

Institute of Pharmaceutical Sciences,

Department Pharmaceutical Technology and Biopharmacy

Universitätsplatz 1

8020 Graz, Austria

Phone: +43 316 380 8888

E-mail: eva.roblegg@uni-graz.at

**SUPPLEMENTARY METHODS**

*Characterization of saliva*

To ensure the comparability of the used saliva samples, we characterized the saliva in terms of whole protein concentration, osmolality and pH. These parameters are essential for maintaining the physiological consistency and protective function of saliva [[1](#_ENREF_1),[2](#_ENREF_2)]. The concentration of the salivary proteins was determined using the Pierce BCA Protein Assay Kit (Life Technologies) according to the manufacturer’s standard protocol, and the color response was measured photometrically at 540 nm. To separate the high-molecular-weight proteins (i.e., mainly mucins), dialysis was performed using a cellulose acetate tube (MW cut-off of 12-14 kDa, Carl Roth); 3 ml of saturated ammonium sulphate solution was added to 10 ml samples at 4°C, and the obtained precipitate was dissolved in 5 ml MQ water. The whole protein content was determined to be 0.67 ± 0.04 mg/ml, of which 0.20 ± 0.02 mg/ml consisted of high-molecular-weight proteins (mostly MUC5B and MUC7). These data are comparable to those reported in the literature [[3](#_ENREF_3)]. The osmolality was measured using an Osmomat O30-D (Gonotec), and the pH, with a pH meter (Lab 860, Schott Instruments). The osmolality of the used samples was determined to be 0.054 ± 0.03 osmol/kg, and the pH varied between 6.8 and 7.4, indicating optimal conditions for simulating the physiological state of saliva [[1](#_ENREF_1),[4](#_ENREF_4)].

**SUPPLEMENTARY FIGURES**


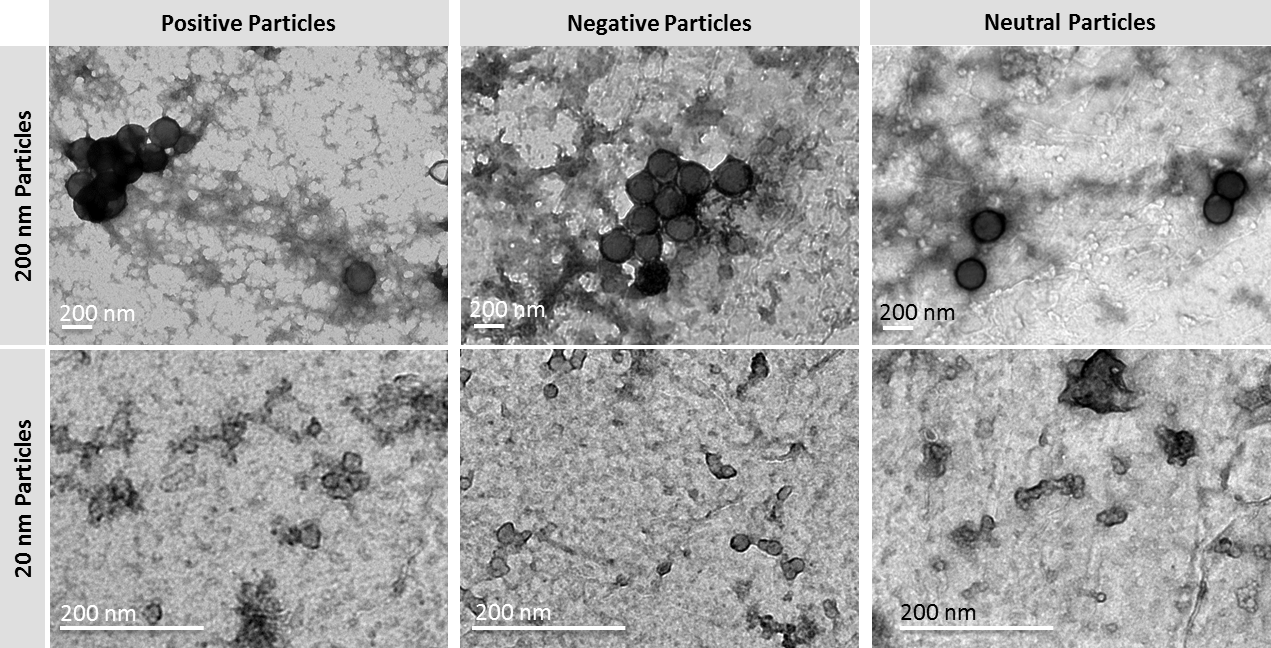


**c)**

**b)**

**a)**

**e)**

**f)**

**d)**

**Fig. S1. TEM images of NPs dispersed in human whole saliva.** The conventional negative staining procedure [[5](#_ENREF_5)] was conducted to visualize the NPs and the mucoglycoproteins. For this purpose, 5 µl of human whole saliva was applied on a glow-discharged carbon-coated copper grid and incubated for 1 minute. 5 µl of a 1% uranyl acetate solution was dropped on the copper grid and incubated for 1 min. a) 200-nm aminated and b) 200-nm carboxylated NPs agglomerated in saliva, while c) 200-nm non-functionalized NPs were only moderately affected. d) 20-nm aminated, e) 20-nm carboxylated and f) 20-nm non-functionalized NPs also agglomerated in saliva.


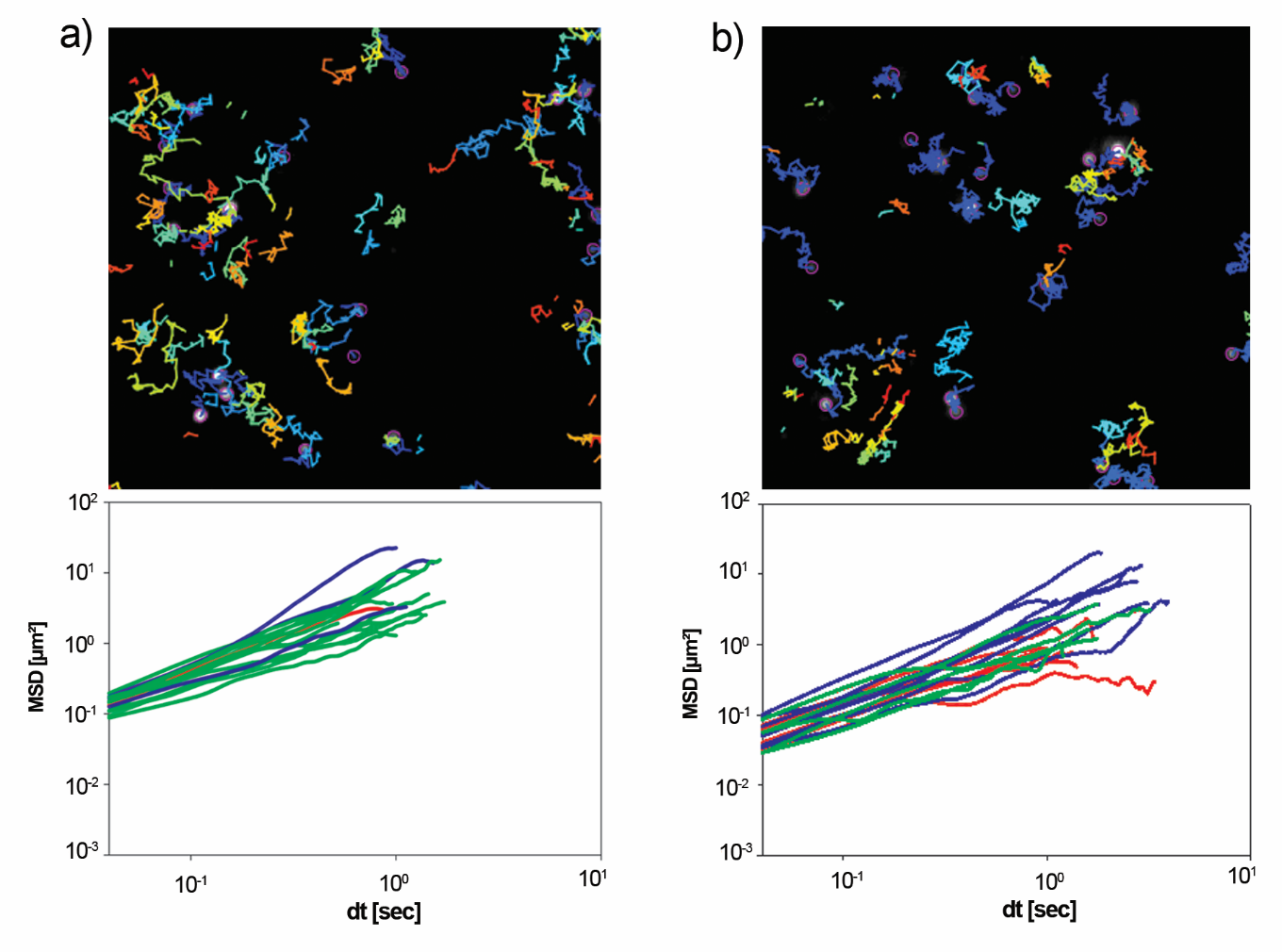
**Fig. S2. NP trajectory studies in water and saliva.** The diffusion of the NPs was recorded by means of NTA at RT. The upper panels depict representative images of NP trajectories and the lower panels show the corresponding MSD plots of individual NPs (n ≥ 20). The colored MSD curves represent normal diffusion (green, α = 1), sub-diffusion (red, α < 1) and super-diffusion (blue, α > 1). a) The 200-nm non-functionalized NPs showed predominantly unhindered Brownian diffusion in water, while b) in saliva also sub- and super-diffusive NP motion was recorded. Moreover, the mobility was 2- to 3-fold slower than in water.

**REFERENCES**

1. Tabak LA, Levine MJ, Mandel ID, Ellison SA (1982) Role of salivary mucins in the protection of the oral cavity. J Oral Pathol Med 11 (1):1-17

2. Schipper RG, Silletti E, Vingerhoeds MH (2007) Saliva as research material: biochemical, physicochemical and practical aspects. Arch Oral Biol 52 (12):1114-1135

3. Lee JY, Chung JW, Kim YK, Chung SC, Kho HS (2007) Comparison of the composition of oral mucosal residual saliva with whole saliva. Oral Dis 13 (6):550-554

4. Humphrey SP, Williamson RT (2001) A review of saliva: Normal composition, flow, and function. J Prosthet Dent 85 (2):162-169

5. Gradauer K, Vonach C, Leitinger G, Kolb D, Fröhlich E, Roblegg E, Bernkop-Schnürch A, Prassl R (2012) Chemical coupling of thiolated chitosan to preformed liposomes improves mucoadhesive properties. Int J Nanomed 7:2523-2534
